# Influence of Gradual Damage on the Structural Dynamic Behaviour of Composite Rotors: Simulation Assessment

**DOI:** 10.3390/ma11122453

**Published:** 2018-12-03

**Authors:** Angelos Filippatos, Albert Langkamp, Maik Gude

**Affiliations:** Institute of Lightweight Engineering and Polymer Technology (ILK), Technische Universität Dresden, 01307 Dresden, Germany; albert.langkamp@tu-dresden.de (A.L.); maik.gude@tu-dresden.de (M.G.)

**Keywords:** gradual damage behaviour, composite rotors, structural dynamic behaviour

## Abstract

Fibre-reinforced composite structures under complex loads exhibit gradual damage behaviour with degradation of effective mechanical properties and change of their structural dynamic behaviour. In case of composite rotors, this can lead to catastrophic failure if an eigenfrequency is met by the rotational speed. The description and simulation analysis of the gradual damage behaviour of composite rotors therefore provides the fundamentals for a first understanding of complex and partially-unpredicted structural phenomena. Therefore, a simulation tool is developed using a finite element model, which calculates the damage-dependent structural dynamic behaviour of selected composite rotors considering both damage initiation and in-plane damage evolution due to a combination of out-of-plane and in-plane loads. Damage initiation is determined using failure criteria, whereas the gradual damage evolution using a validated continuum damage mechanics model. Numerical results are compared with experimental results for rotor-typical stress states to assess the model quality, which could be later used for damage identification approaches.

## 1. Introduction

The development of innovative and efficient damage identification methods is a complex engineering challenge. It requires an interdisciplinary approach that uses experimental, simulation and information-based methods, which encompass the material and structural behaviour. In this context, the understanding of the gradual damage behaviour and of the corresponding structural vibration response of composite structures plays a central role.

A general definition of damage in composite structures as it relates to damage identification methods is given by Sohn [1] as changes in the geometry or in the material properties of a composite structure that affect its current or future performance. In this context, in order to accurately define damage, a comparison between two different structural states has to be performed, one of which is assumed to represent the initial and undamaged state. Therefore, a physical and reliable simulation of the composite damage and the occurring structural phenomena is an important prerequisite for a successful damage identification.

### 1.1. Motivation

An important issue within damage identification is the complexity of composite structures combined with a vast amount of possible combinations of failure modes, damage extents and their positions. Therefore, a common approach for the development of damage identification methods is the application of machine learning algorithms, in which these algorithms are trained to learn datasets containing representative damage configurations [2]. However, in most instances the generation of such learning sets is not realistic in an experimental way due to prohibitive time and cost factors [3,4]. Therefore, a solution to this problem is the development of reliable simulation models for the generation of learning sets considering only a minimum amount of experimental data for fitting purposes [5]. In this context, phenomenological-based damage models capable of predicting different diffuse and discrete damage phenomena have been further developed to describe the progressive damage of composite materials until final failure [6].

### 1.2. State-of-the-Art

The stress–strain behaviour of fibre-reinforced composites can be modelled by constitutive relations based on the generalized Hooke’s law in the area of small displacements and under the limits of damage initiation, which is normally the first-ply inter-fibre failure [7]. For the initial failure of fibre-reinforced composites under static, cyclic and highly-dynamic stress, descriptive damage models exist based on the failure criteria of Hashin, Puck and Cuntze [8,9,10]. Furthermore, for the theoretical description of the gradual damage behaviour of composite materials, these advanced models have been further developed and confirmed. The description of damage as a continuous process forms the basis for the further analysis of the gradual damage behaviour of composites and the corresponding damage-dependent dynamic behaviour.

### 1.3. Gradual Damage Behaviour of Composite Materials

For the description of the gradual damage behaviour of composites, caused by operational loads or unpredicted loads such as impacts, verified damage mechanics models for composite materials are already available [11]. Typical failure modes for composite rotors are mainly inter-fibre failure from in-plane loads as has been shown in [12] and a mixture of inter-fibre failure, delaminations and fibre failure from unexpected impact loads [13]. However, the application of these damage mechanics models for rotor-typical loading conditions under consideration of the gradual damage behaviour and the resulting structural dynamic behaviour has not been thoroughly investigated.

An example of such an approach can be a phenomenological damage mechanics model [6] combined with a simulation model [14]. Based on such a model, the gradual damage analysis and the resulting dynamic behaviour of composite rotors can be described. In particular, some first qualitative results have been presented [12,15] for typical fibre architectures which have previously been extensively investigated with their material properties characterised in [16,17].

### 1.4. Damage Initiation Modes

For the calculation of the damage initiation under complex loading conditions, a reliable damage mechanics model was applied that considers the material symmetries by the application of invariants [10,18]. A unidirectional lamina was homogenised to a “material” and separated strength criteria are allocated to each failure mode and to each associated basic strength. The Failure Mode Concept (FMC) focuses on two expressions of the theoretical prediction of failure in composites. The first expression is the derivation of failure conditions for a unidirectional lamina with the prediction of initial failure of the embedded lamina. The second expression is the treatment of a non-linear, progressive failure of three-dimensionally stressed laminates until final failure [10].

**Figure 1:**
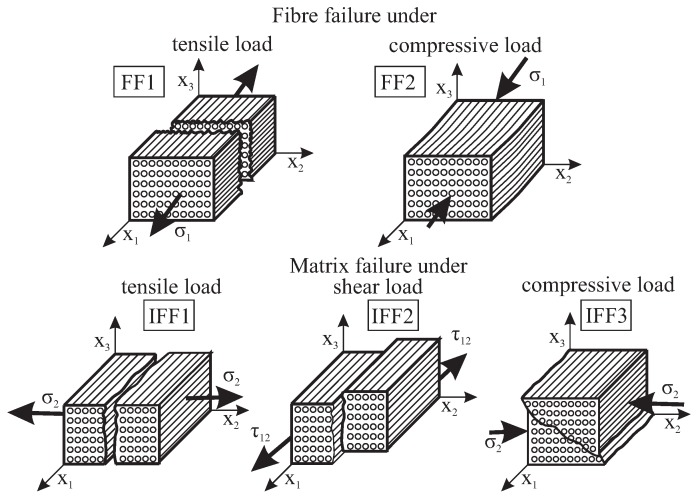
View of the failure modes of a brittle, transversally-isotropic material based on the Failure Mode Concept [18].

According to the FMC, five different failure modes can be identified, as shown in Figure 1. At low load levels, there is formation of three different matrix failure types, IFF1, IFF2 and IFF3, under tensile stress $\sigma_{2}>0$, shear stress $\tau_{21}$ and compressive stress $\sigma_{2}<0$, respectively. Increasing stresses result in extended matrix failure and in the formation of delaminations. With further increase of tensile stress $\sigma_{1}>0$ and compressive stress $\sigma_{1}<0$, resulting in extensive distributed damage, there is the formation of tensile FF1 or compressive FF2 fibre failures, respectively [18].

For every single failure mode, an equivalent stress applying the FMC exists with $\sigma_{equ}^{*}$ with * = ∥σ,∥τ,⊥σ,⊥τ,⊥∥ based on formulated invariants and the strengths from the defined material efforts $Eff^{*}$. The used formulations for the initiation of damage as well as the final failure are based on the following equations of single efforts [10]:

(1) $$\begin{array}{c} Eff^{∥\sigma }=\frac{\sigma_{1}}{R_{∥}^{t}}\;,\;Eff^{∥\tau }=-\frac{\sigma_{1}}{R_{∥}^{c}}\\ Eff^{⊥\sigma }=\frac{\sigma_{2}}{R_{⊥}^{t}}\;,\;Eff^{⊥\tau }=-\frac{\sigma_{2}}{R_{⊥}^{c}}\;,\;\\ Eff^{⊥∥}=-\frac{|\tau_{21}|}{\left(R_{⊥∥}-\mu \sigma_{2}\right)} \end{array}$$

The final effort $Eff^{tot}$ results from a serial connection of the single efforts

(2) $$\begin{array}{c} Eff^{tot}=({\left(Eff^{∥\sigma }\right)}^{m}+{\left(Eff^{⊥\sigma }\right)}^{m}+\\ +{\left(Eff^{∥\tau }\right)}^{m}+{\left(Eff^{⊥\tau }\right)}^{m}+{\left(Eff^{⊥∥}\right)}^{m}{)}^{\frac{1}{m}} \end{array}$$

using the rounding coefficient *m* [18].

### 1.5. Damage Evolution Approach

For the description of the non-linear gradual damage behaviour, damage evolution laws are then applied, which are based on the constitutive equations for a damaged layer using the applied continuum damage mechanics model. Damage evolution laws have been developed based on FMC using a damage tensor *D*, which is chosen according to identified degradation mechanisms [6].

**Figure 2:**
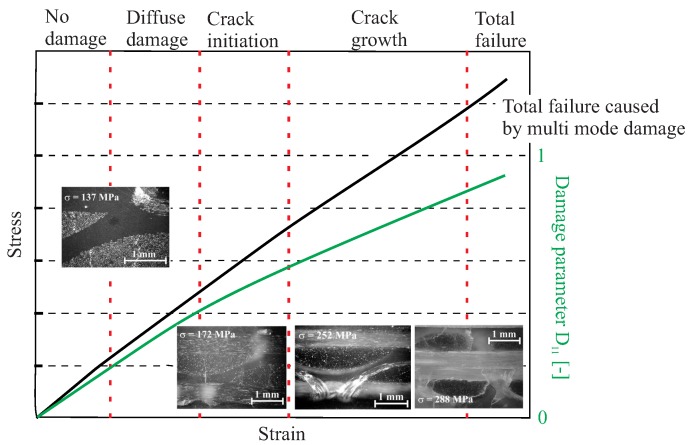
Schematic example of the gradual damage behaviour of a $\left[0^{{}^{\circ}}/90^{{}^{\circ}}//90^{{}^{\circ}}/0^{{}^{\circ}}\right]$ glass fibre-reinforced epoxy under homogeneous tensile loading [19].

A typical example of the gradual damage behaviour of a $\left[0^{{}^{\circ}}/90^{{}^{\circ}}//90^{{}^{\circ}}/0^{{}^{\circ}}\right]$ glass fibre-reinforced epoxy is shown in Figure 2. The specimen undergoes a homogeneous tensile loading, which results in a gradual material degradation. The stress–strain behaviour of the specimen can be viewed with regard to the $D_{11}$ damage parameter affecting the $E_{1}$ stiffness degradation in the longitudinal fibre direction. Five damage states are also identified for the specific composite configuration, illustrating the gradual damage behaviour of composites [19].

For the description of the gradual damage behaviour, fracture mode-specific evolution equations are predestined, which capture both the stiffness degradation and coupled degradations from the direction-dependent stiffness. These relationships were investigated and described, in particular, for textile-reinforced glass epoxy resins [20]. Based on these findings, further approaches were developed with regard to their implementation into the finite element method [21].

### 1.6. Delamination Mechanisms

In addition to the failure and damage processes mentioned above, which are typically described by continuum mechanics models, delamination is the main out-of-plane failure mode. A variety of methods have been developed for the analysis of the damage behaviour of composites under impact loads causing delamination until final failure [22]. Such methods cover a detailed representation of delamination using cohesive models or the virtual crack closure-integral method, in particular for Mode I and Mode II delaminations [23,24]. Mode III is often not modelled in composites, as it rarely occurs and it is experimentally difficult to characterise. Furthermore, simulation techniques for multiple failure modes, including delamination, have been successfully applied in a simpler, yet reliable method for multiple composite structures [25]. These methods follow material degradation rules by the simulation of the progressive damage propagation at composite structures.

**Figure 3:**
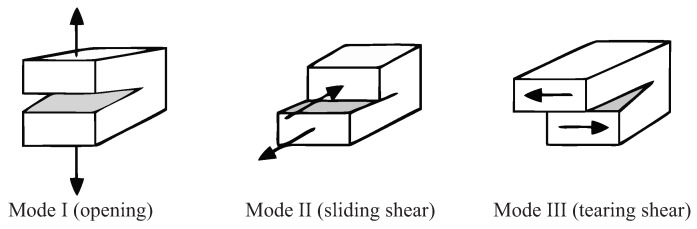
Fracture modes: Mode I with tensile delamination (**left**), Mode II with sliding shear delamination (centre), and mode III with tearing shear delamination (**right**).

Commonly, the delamination process in composites is described in terms of delamination initiation and delamination propagation, caused by the out-of-plane tensile stress $\sigma_{3}$ and shear stresses $\tau_{13}$, $\tau_{23}$ [26]. Specifically, two different mechanisms of delamination initiation exist, namely, tensile delamination induced by out-of-plane tensile stress $\sigma_{3}$, and shear delamination under $\tau_{13}$, $\tau_{23}$, that can initiate the delaminations and their propagation, as shown in Figure 3 for three delamination modes.

Delamination initiation is mainly treated within the framework of continuum damage mechanics approaches, using quadratic delamination criteria, as shown in Equation (3),

(3) $${\left(\frac{\tau_{13}}{R_{13}^{s}}\right)}^{2}+{\left(\frac{\tau_{23}}{R_{23}^{s}}\right)}^{2}+{\left(\frac{\sigma_{3}^{t}}{R_{3}^{t}}\right)}^{2}+{\left(\frac{\sigma_{3}^{c}}{R_{3}^{c}}\right)}^{2}=1$$

where $R_{3}^{t}$ is the tensile interlaminar normal strength, $R_{3}^{c}$ the compressive interlaminar normal strength, $R_{13}^{s}$ the interlaminar shear strength for $\tau_{13}$ shear stresses and $R_{23}^{s}$ the interlaminar shear strength for $\tau_{23}$ shear stresses [27,28,29].

Different methods have been developed to model delamination within a finite element analysis [22,30]. Firstly, for Mode I and Mode II delaminations, a detailed representation of delamination can be achieved using cohesive models or the virtual crack closure-integral method [23,24]. Mode III is often not modelled in composites, as it rarely occurs and it is experimentally difficult to characterise. Secondly, to model the delamination in a simple, yet reliable method, a different approach has been adopted for several composite structures [25,31]. This approach follows specific structural degradation rules resulting in the degradation of the effective mechanical properties in the delaminated area, and accordingly, the values of ${\tilde{Q}}_{ij}$ [30,32].

### 1.7. Aim and Outline of the Paper

The aim of the paper is to develop a parametric simulation model capable of depicting the structural dynamic behaviour of composite rotors under propagating damage due to a combination of out-of-plane and in-plane loads.

An elementary rotor geometry is selected with a representative fibre architecture for a Cartesian-orthotropic material behaviour. Based on experimental results and their physically-based interpretations [33], a finite element model is developed, which is used for a parametric simulation-based study of the damage-dependent structural dynamic behaviour. The numerically obtained vibration responses are compared with experimental results for rotor-typical stress states.

## 2. Selection of Representative Fibre Architecture for Composite Rotors and Manufacturing

For the experimental investigation of the damage behaviour of composite rotors and their resultant dynamic behaviour, a characterisation of failure-tolerant fibre-reinforced composites is performed. In particular, a multi-ply multi-axial fabric is selected resulting in an in-plane Cartesian-orthotropic behaviour.

### 2.1. Multi-Ply Multi-Axial Fabric

**Figure 4:**
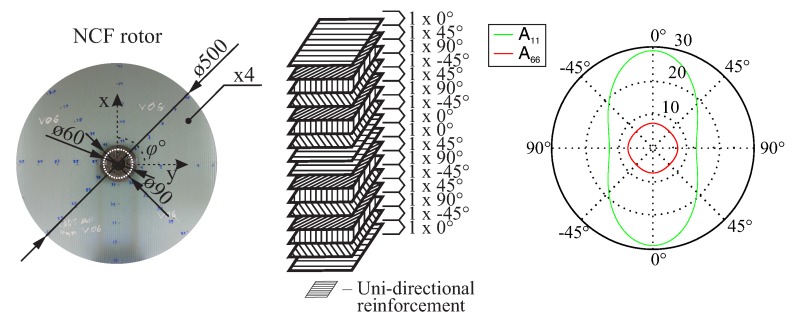
Geometry of a NCF rotor (**left**) with the lay-up (**centre**) and the corresponding homogenised directional stiffness properties A${}_{11}$, A${}_{66}$, resulting in an in-plane Cartesian-orthotropic behaviour (**right**).

The selected fibre architecture is composed of a glass-fibre, non-crimp, multi-ply and multi-axial fabric (NCF) [34,35]. The fabric reinforcement has an area density of 1.90 kg/m^2^ and a ply thickness of 1 mm. The composite lay-up consists of four such fabrics $[\left(0^{{}^{\circ}}/-45^{{}^{\circ}}/90^{{}^{\circ}}/+45^{{}^{\circ}}\right)/(-45^{{}^{\circ}}/90^{{}^{\circ}}$$/45^{{}^{\circ}}/0^{{}^{\circ}})/\left(0^{{}^{\circ}}/-45^{{}^{\circ}}/90^{{}^{\circ}}/45^{{}^{\circ}}\right)/\left(-45^{{}^{\circ}}/90^{{}^{\circ}}/45^{{}^{\circ}}/0^{{}^{\circ}}\right)]$, as shown in Figure 4, resulting in a total laminate thickness of 4 mm and in an in-plane orthotropic behaviour. The inner and outer diameter of the rotor are 60 mm and 500 mm, respectively.

This material was extensively investigated in previous research projects, where the results indicated a gradual damage behaviour [17]. The manufacture of the composite rotors is carried out using the vacuum-assisted resin transfer moulding (VARTM) process [17].

### 2.2. Quality Assessment of Manufactured Rotors

In order to determine the thickness variation, quality assessment is performed on the manufactured NCF-rotors, using the optical marker recognition system Tritop and the digitizing system Advanced Topometric Optical Sensor (Atos) from the company GOM. Multiple measurements are taken for each rotor and combined using the system Tritop, in order to minimise the error by the data combination. Each single 3D scanning of the entire test object has a spatial point-to-point distance of approximately 0.27 mm by using a camera system with an optical resolution of 1280×1024 pixel, enabling the measurement of small, local deformations and manufacturing defects at the rotor surface.

**Figure 5:**
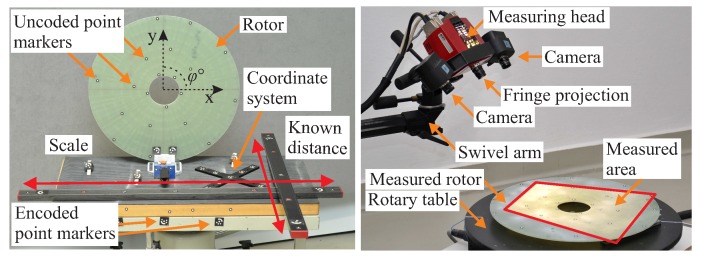
Arrangement of the rotors for the creation of reference point clouds using the Tritop system (**left**), and for the measurement of the manufacturing variations on a rotating table using the Atos system (**right**).

The rotor is placed on a rotary table and it is measured with a high resolution of approximately 30 μm, as shown in Figure 5.

For the determination of the rotors thickness variation, the acquired experimental data are further analysed using parametric fitting, in order to determine the shape of the rotors. A quadratic polynomial curve is used as fitting equation of the type

(4) $$\begin{array}{cc} y\left(x\right)=q_{1}x^{2}+q_{2}x+q_{3}\;,\; & x\in [35,250]. \end{array}$$

**Figure 6:**
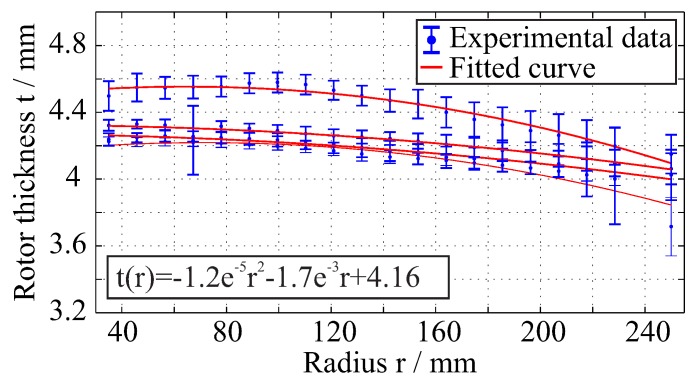
Thickness variation for multiple NCF rotors based on experimental data from the Atos system.

Specifically, 36 evenly distributed rotor sections are selected with approximately 650 data points each. Then, the mean thickness value and the standard deviation is calculated along the radius of the rotor for 20 evaluation points, and a quadratic polynomial curve is fitted for each manufactured rotor. The resulting manufacturing variations of the rotors caused by the internal pressure are presented in Figure 6.

According to the experimental results, the thickness varies along the rotor radius. The applied inner pressure of 4 bar in the manufacturing mould slightly deformed the upper part of the mould. This deformation of the VARTM tool resulted in an increase of the volume of the manufacturing mould. This volume expansion is reflected in the dimensions of the manufactured rotors. Compared to the nominal rotor thickness of 4 mm for the rotors, a deviation of the thickness of the manufactured rotors is observed locally up to 0.5 mm.

## 3. Development of a Parametric Simulation Model

A simulation model is developed, which predicts the damage-dependent structural dynamic behaviour of the investigated composite rotors under consideration of both the damage initiation and the in-plane damage evolution from a combination of out-of-plane and in-plane loads. Within this model, the damage initiation is determined by means of the failure criterion. The gradual damage evolution can be described with a validated continuum damage mechanics model, which captures the degradation processes after considering the failure modes and respective damage coefficients.

In addition, the simulation results are validated with experimental results from intact and damaged rotors regarding the gradual damage behaviour and the corresponding damage-dependent dynamic response [33].

**Figure 7:**
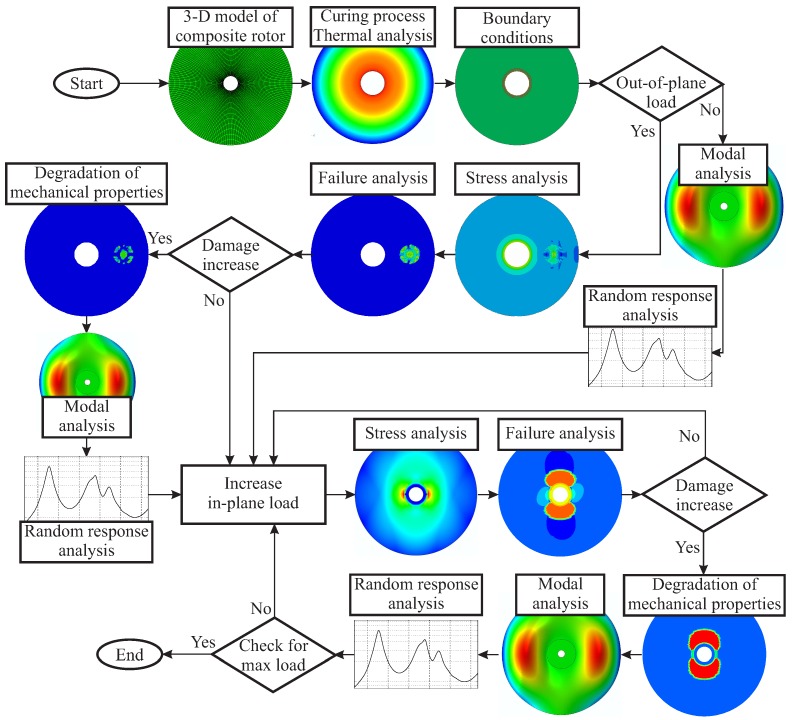
Flowchart for the progressive failure damage analysis for a composite rotor under out-of-plane and in-plane loading.

For the development of a parametric simulation model, the finite element (FE) software Abaqus is used, and a parametric Python script is implemented for the investigation of the damage-dependent dynamic behaviour. This enables the generation of a FE model with parameters that define the position and magnitude of the out-of-plane load. Subsequently, a finite element analysis (FEA) is performed, and a detailed investigation of the gradual damage evolution as well as the resulting dynamic behaviour of the rotors is conducted. The flow chart of the developed simulation approach is shown in Figure 7.

**Figure 8:**
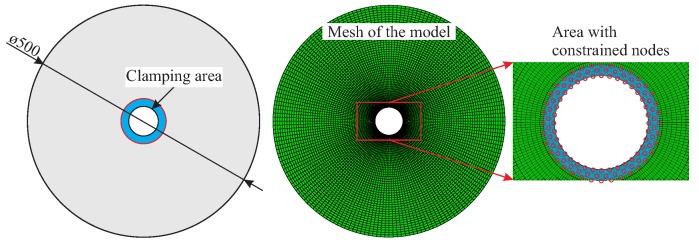
Schematic illustration of the rotor geometry (**left**); generated FE-mesh for the investigated rotors, with one element in the thickness direction and an approximate element thickness of 5 mm (**middle**); and the boundary conditions of the FE model (**right**).

For each type of rotor, the geometry is defined according to the measured averaged thickness of the manufactured rotors, which was previously experimentally determined. Specifically, the preform thickness and the internal pressure-induced deflection of the mould are taken into account. Manufacturing imperfections such as the deviation of the fibre orientation or local resin pockets are not modelled. Material parameters determined in previous investigations are also retrieved [17]. The model is meshed using continuum three-dimensional, 8-node linear brick solid elements with incompatible modes of type C3D8I with approximately a total number of 15,500 nodes and 7500 elements as shown in Figure 8. Based on the selected type of element, results from previous investigations as well as the comparison of the numerically estimated to the experimentally measured modal properties, one element in the thickness direction is sufficient for this kind of analysis and the desired resolution. The quality of the mesh plays a very important role both at the damage prediction as well as to the eigenfrequency calculations. In order to identify an optimal size of the elements that result in sufficient good results, a convergence investigation for the first eigenfrequencies has been performed at preliminary investigations. This investigation showed that with this element size we achieve a convergence. A qualitative comparison between experimentally and numerically determined damage states took place. A further more detailed investigation for the mesh quality will be taken into consideration in future investigations for similar rotors with a variable thickness along the radius. For the boundary conditions of the model, the degrees of freedom located at the inner area of the rotor are constrained in order to simulate the clamping area. In the boundary nodes all the degrees of freedom are constrained. The clamping is modelled by selecting the nodes located at the inner area of the rotor, and then by setting the translational degrees of freedom of these nodes in the out-of-plane direction of the rotor as well as the respective rotational degrees of freedom to zero, as shown in Figure 8.

A user-defined field subroutine (USDFLD) [21] is included to model the damage initiation and the gradual damage behaviour of NCF composite rotors. This subroutine enables the description of the damage initiation and propagation, with respect to previously developed phenomenological damage models [20]. In this work, the modelling of delamination using a reduction of effective mechanical properties is followed with regard to Equation (3). This technique, although simple, is considered as sufficient, according to experimentally gathered information from ultrasonic testing and its comparison to the simulation results.

The considered loads are derived from previous experimental investigations [33]. The out-of-plane load, which creates the initial damage, is modelled as a distributed load at the nodes included in the contact area between impactor and rotor [33]. The rotor run-up is modelled as rotational velocity steps of 1000 rpm in a range from 8000 rpm through 14,500 rpm for a NCF rotor based on previous experiments [33]. The applied increasing body forces cause the increase of complex states of stress as well as the initiation and the resulting damage evolution.

In order to consider the manufacturing-relevant residual stresses of the rotor, the curing process is simulated as a pre-stress effect, where a thermal static analysis is included with a temperature change of ΔT= 60 K. Subsequently, the stress fields are calculated and used for the failure analysis as well as for the calculation of the damage initiation. Based on the calculated data, the damage evolution at each layer is estimated and the corresponding effective material properties are included in the model. The estimated developed damage is subsequently transferred as starting state for the next loading step.

For each estimated damage state, the damage-dependent dynamic response of the rotor is calculated using a numerical modal analysis and, approximately, the first 20 eigenmodes are estimated for a frequency range from 20 Hz through 1000 Hz. Subsequently, a random response analysis is performed for the calculation of the power spectral densities at selected degrees of freedom [33]. The excitation is simulated as an uncorrelated band-limited white noise in the frequency range of interest.

The consideration of damping in commercially available software programs is state of the art for classical isotropic monolithic materials. However, the direction-dependent damping coefficients of anisotropic materials are not supported. To overcome this deficiency, a post-processing feature has been developed in Matlab, able to calculate the modal loss factor of anisotropic fibre-reinforced composite structures and is here also implemented [36,37]. The model includes the anisotropic material damping by an extension of the FEM using the strain energy concept [38,39]. The dynamic response is extracted at 128 degrees of freedom corresponding to the measurement points of previous experimental modal analysis [33].

The respective damage-dependent change of the material damping is not considered due to the missing material damping values. The estimation of the damage- and direction-dependent damping coefficients of anisotropic materials is still a challenge, which is already in the scientific focus [40], but still has to be addressed in future investigations.

The described simulation analysis is performed sequentially for each defined rotational velocity. The simulation delivers numerical results for the local stresses of the rotor, for the resulting efforts and the respective failure modes as well as for the dynamic response of the rotor.

## 4. Assessment of the Parametric Simulation Model

A comparison between the experimental and the numerical results is performed for the validation of the simulation approach [33]. First, the simulation model is assessed with regard to the damage state and the damage evolution. Then, the dynamic vibration response is compared and the modal dynamic properties, i.e. the mode shapes, the eigenfrequencies and the modal loss factor are compared for multiple structural states. Finally, the dynamic behaviour is considered under the effect of the evolving damage and a comparison between experimental and numerical change of eigenfrequencies is conducted.

### 4.1. Damage State and Damage Evolution

**Figure 9:**
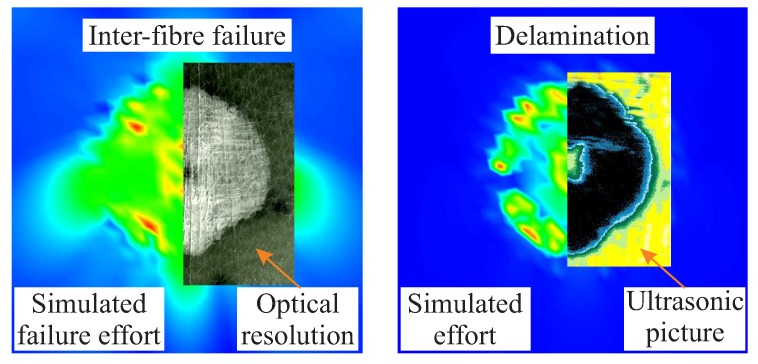
Numerically estimated and experimentally determined damage after an out-of-plane compression load of 16 kN and the resulting inter-fibre effort (**left**) as well as the delamination (**right)**.

A comparison of the damage effect of an out-of-plane compression load of 16 kN an area between experimental and numerical results reveals a similar damage extent as well as the same type of damage, as shown in Figure 9. The contact area was simulated with a diameter of 10 mm, based on experimental results [33]. The same main failure modes appear both in the model and in the experiments, which are inter-fibre failure, fibre-matrix shear failure and the formation of delamination. Overall, the investigation of the damage state at different applied compression loads confirms a good qualitative agreement of the damage extent according to experiments and simulation.

**Figure 10:**
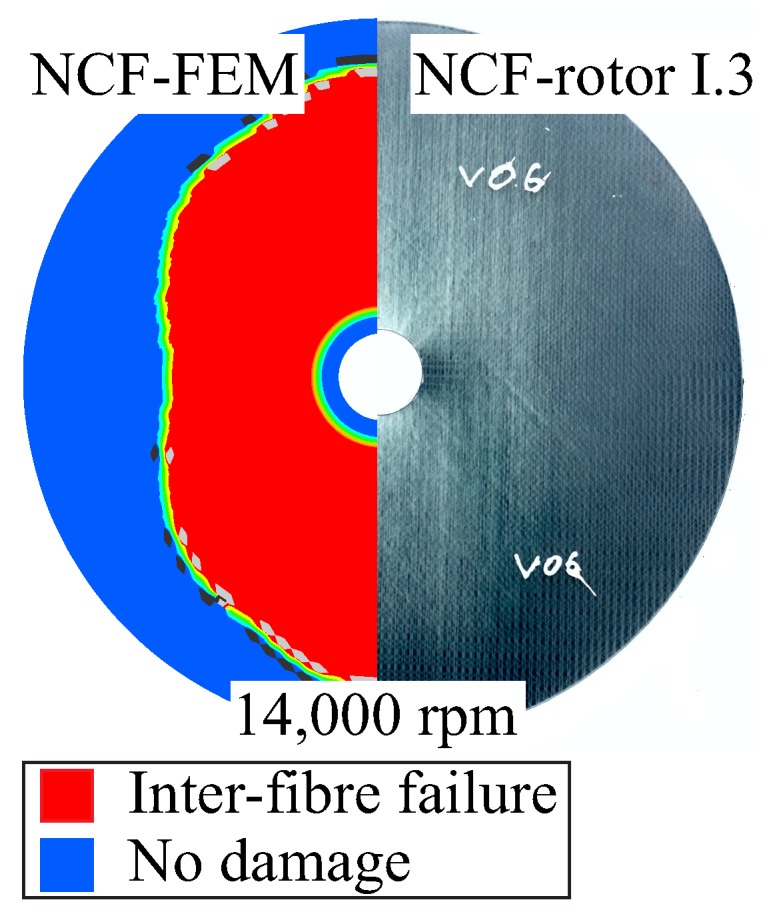
Numerically and experimentally determined inter-fibre failure for an in-plane load of 14,000 rpm.

The damage initiation and propagation due to an in-plane load under a rotational velocity is shown in Figure 10. The damage evolution is not-symmetric due to the selected fibre architecture and the resulting in-plane Cartesian orthotropic material behaviour. A qualitative accordance is observed with regard to the damage extension as well as the main failure modes that are apparent, which are inter-fibre failure due to tensile and shear stresses.

### 4.2. Modal Properties

With regard to previous experiments [33], the developed model provides all the necessary information for the dynamic behaviour of the investigated composite rotors, mainly the mode shapes, the eigenfrequencies, the consequent change of eigenfrequencies due to damage increase, the modal loss factor and the single as well as the mean power spectral density. For the model validation, the experimental and numerical properties are compared for a frequency range from 20 Hz through 2000 Hz, including 16 experimentally measured eigenmodes.

#### 4.2.1. Mode Shapes of Intact Rotors

**Figure 11:**
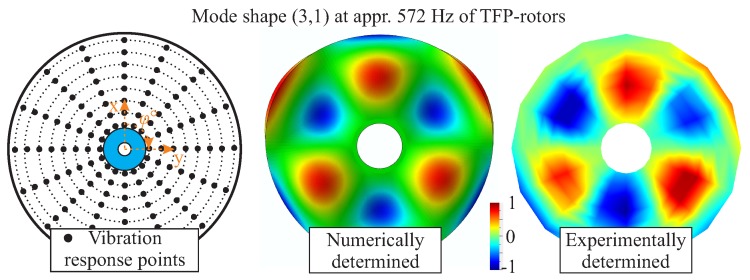
Comparison between the experimentally identified and numerically calculated mode shapes of an undamaged rotor.

A qualitative comparison between a calculated and an experimentally determined mode shape (3,1) for an intact NCF-rotor is shown in Figure 11. The calculated mode shape corresponds with the experimentally measured one, providing a first qualitative verification of the simulation model.

**Figure 12:**
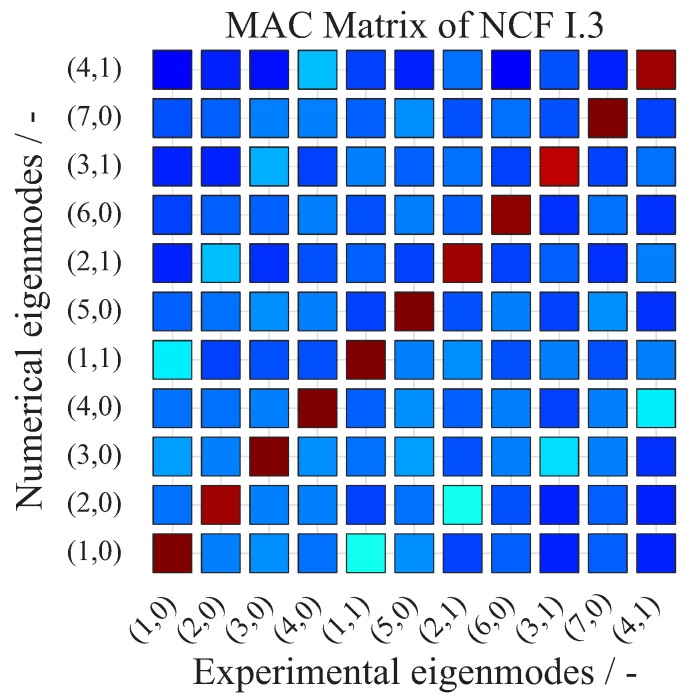
Comparison of measured and calculated mode shapes using the MAC for the NCF rotor I.3.

For the quantitative evaluation of the developed model with respect to the mode shapes, the Modal Assurance Criterion (MAC) is applied, which yields a good statistic indicator and a degree of consistency between mode shapes. The MAC is calculated as

(5) $$MAC=\frac{{\left(x_{exp}^{T}x_{num}\right)}^{2}}{\left(x_{exp}^{T}x_{exp}\right)\left(x_{num}^{T}x_{num}\right)}$$

which forms the linear dependency of two modal vectors $x_{exp}$, $x_{num}$. Each modal vector $x_{exp}$, $x_{num}$ is the vector of a mode shape with a length of n=128 measured and calculated points, respectively. A MAC value of unity represents a collinearity of the two vectors, which indicates fully consistent mode shapes. In contrast, a MAC value of zero indicates the orthogonality of the two eigenvectors, which means that the modes are not consistent. A tolerance limit of 70% is usually acceptable for a consistent correlation between the investigated mode shapes [41]. A high agreement of the mode shapes is observed for the 11 identified EFs for the NCF I.3 rotor, as it is shown in Figure 12, as well as for the other investigated rotors.

#### 4.2.2. Modal Loss Factor

**Figure 13:**
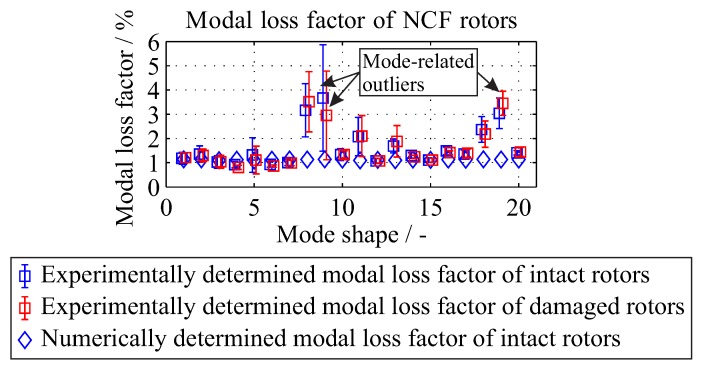
Comparison between the experimentally identified and numerically calculated modal loss factors for the investigated rotors.

The comparison of the numerical and experimental results of the modal loss factor is shown in Figure 13. The quantitative comparison showed a good accordance with deviations up to 4%. The differences between experimental and calculated modal loss factors are due to the contribution of friction-caused damping at the clamping area.

A mean modal loss factor of 1.61% and 1.12% is experimentally and numerically determined. Due to the small changes, a constant modal loss factor of 1.12% will be used for the simulation. For more complicated structures where the mode shapes are substantially different, a variance of the modal loss factor is expected and in this case a vector array should be used.

For the damaged states, the modal loss factor remains almost constant, with a mean value of 1.74% with a standard deviation of 0.33%, based on experimental data from all investigated rotors. These values are similar to the modal loss factor determined previously for the undamaged states. Therefore, the modal loss factor from the undamaged state can be used as constant values for both damaged and undamaged states, although it is expected that the damaged states should have an increased modal loss factor due to friction in the interface of the inter-fibre cracks. The possible friction in the interface of the inter-fibre cracks can influence the damping behaviour of the investigated composite rotors. The selected method of extracting the modal damping ratio provides a first assessment of the global frequency-dependent damping [42]. When a further more detailed damping estimation is required, then an estimation of the spatial distribution of the damping properties using a dynamic mechanical analysis could be applied [40]. In this way also damage-caused increase of the anisotropic material damping can be investigated, which originates from dry friction in fibre-reinforced polymer-based composites [43].

### 4.3. Change of Eigenfrequencies Due to Damage Increase

**Figure 14:**
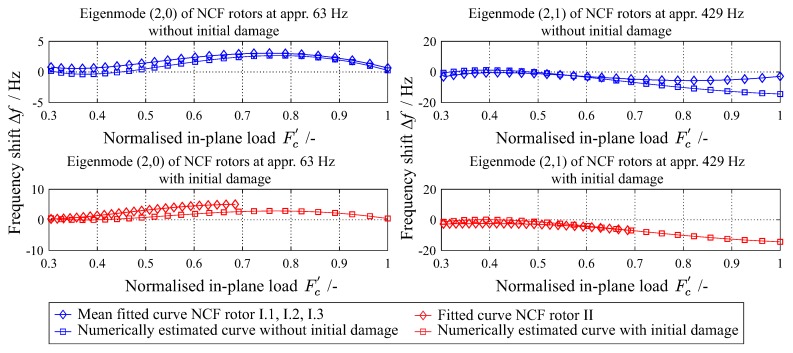
Experimentally and numerically determined change of the eigenfrequency for two eigenmodes of a rotor, with and without initial damage.

Figure 14 shows the experimentally and numerically determined eigenfrequencies of a NCF rotor and two eigenfrequencies, the (2,0) and (2,1) are exemplary presented. Specifically, one with a non-monotonic change of the eigenfrequency (left) and one with a monotonic decrease of the eigenfrequency (right) is observed.

The relation between applied in-plane loads and the corresponding shift of eigenfrequencies due to damage increase is investigated for five different damage sequences. For each damage sequence, a total of 16 eigenfrequencies are investigated. For each type of rotor, a normalised in-plane load $F_{c}^{{}^{\prime }}$ is introduced,

(6) $$F_{c}^{{}^{\prime }}={\left(\frac{n_{rpm}}{n_{rpm}^{max}}\right)}^{2}$$

where each rotational velocity $n_{rpm}$ is divided from the maximum achieved rotational velocity $n_{rpm}^{max}$ for each sequence.

**Figure 15:**
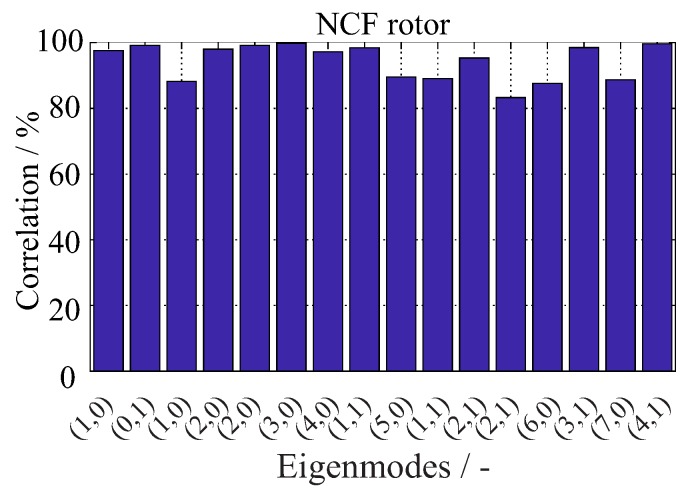
Statistical comparison between experimentally identified and numerically calculated changes of eigenmodes, using the correlation measure, for an undamaged rotor.

A quantitative assessment of the model’s performance is conducted with regard to eigenfrequency change. The correlation is selected as a statistical criterion for this assessment. The correlation is calculated between the mean fitted curves of the experimental ($EF_{exp}$) and numerical ($EF_{num}$) eigenfrequencies, and the results are shown in Figure 15. There is approximately more than 80% correlation between experiment and simulation for all investigated eigenfrequencies.

## 5. Conclusions

Based on experimental results and their physically-based interpretation [33], a parametric simulation model is developed and verified, which numerically determines the damage-dependent structural dynamic behaviour of the investigated composite rotors. This model can be later used, e.g., for the generation of input data required for different damage identification methods. A very good agreement between measurements and simulation results is identified and validated for rotor-typical stress states. Overall, the good compliance of experimental measurements and numerical simulations shows that the proposed numerical approach can provide a reliable way for simulating the dynamic behaviour of composite rotors with a complex fibre architecture under different structural conditions.

The deviation between experimentally and numerically determined results is reasoned by production-related features, such as the thickness variation of the manufactured rotors, preform thickness variations, small changes to fibre orientation and local resin pockets. On the one hand, a better manufacturing process would result in a higher reliability and therefore in a better accuracy of the models. On the other hand, an exemplar-specific modelling and simulation approach of the rotors could also improve the accuracy of the models and could be applied in the field of digital twins of composite rotors.

## Abbreviations

The following abbreviations are used in this manuscript:

FMC	Failure Mode Concept
IFF	Inter-fibre failure
FF	Faibre failure
NCF	Non-crimp fabric
VARTM	vacuum-assisted resin transfer moulding
ATOS	Advanced topometric optical sensor
FE	Finite element
USDFLD	user-defined field subroutine
MAC	Modal assurance criterion
EF	Eigenfrequency
